# Xapuri virus, a novel mammarenavirus: natural reassortment and increased diversity between New World viruses

**DOI:** 10.1038/s41426-018-0119-9

**Published:** 2018-06-29

**Authors:** Jorlan Fernandes, Alexandro Guterres, Renata Carvalho de Oliveira, John Chamberlain, Kuiama Lewandowski, Bernardo Rodrigues Teixeira, Thayssa Alves Coelho, Charle Ferreira Crisóstomo, Cibele Rodrigues Bonvicino, Paulo Sérgio D’Andrea, Roger Hewson, Elba Regina Sampaio de Lemos

**Affiliations:** 1 Laboratory of Hantaviruses and Rickettsiosis, Oswaldo Cruz Foundation, Oswaldo Cruz Institute, Rio de Janeiro – RJ, 21040-360 Brazil; 2 National Infection Service, Public Health England, Porton Down, Salisbury, Wiltshire SP4 0JG UK; 3 Laboratory of Biology and Parasitology of Wild Mammals Reservoirs, Oswaldo Cruz Foundation, Oswaldo Cruz Institute, Rio de Janeiro – RJ, 21040-360 Brazil; 4 Federal Institute of Acre, Rio Branco – AC, 69900-640 Brazil; 5 Postgraduate Program in Biodiversity and Health, Oswaldo Cruz Foundation, Oswaldo Cruz Institute, Rio de Janeiro – RJ, 21040-360 Brazil; 6 Nacional Cancer Institute, Rio de Janeio – RJ, 20230-130 Brazil

## Abstract

Mammarenavirus RNA was detected in Musser’s bristly mouse (*Neacomys musseri*) from the Amazon region, and this detection indicated that rodents were infected with a novel mammarenavirus, with the proposed name Xapuri virus (XAPV), which is phylogenetically related to New World Clade B and Clade C viruses. XAPV may represent the first natural reassortment of the *Arenaviridae* family and a new unrecognized clade within the Tacaribe serocomplex group.

## Introduction

Arenaviruses are bi-segmented ambisense RNA viruses hosted by rodents, bats, snakes, shrews, and ticks^1–3^. The *Arenaviridae* family currently comprises 41 viral species, classified into three genera, *Mammarenavirus* (35 species), *Reptarenavirus* (five species), and *Hartmanivirus* (one specie)^4^. Each of the two arenavirus RNA segments encodes genes for two non-overlapping reading frames in ambisense polarity: the large (L) genomic segment for the viral RNA-dependent RNA polymerase (RdRp or L protein) and a zinc-binding matrix protein (Z protein), whereas the small (S) genomic segment encodes for the nucleocapsid protein (NP) and glycoprotein precursor (GPC), which are post-translationally processed into the envelope proteins G1 and G2 and the stable signal peptide (SSP)^1,5^.

Mammarenaviruses were also classified into two groups according to their genomic features and antigenic properties: the Old World Lassa-Lymphocytic choriomeningitis virus (LCMV) serocomplex, including viruses from Africa and, recently, Asia; and the New World Tacaribe serocomplex, formed by viruses indigenous to the Americas^1,5–7^.

Despite the increased number of Old World viruses characterized in recent years^3^, New World mammarenaviruses remain the most genetically diverse viral group within the family, composed of 18 species divided into four lineages: Clade A, Clade A-recombinant (Clade D), Clade B, and Clade C, according to their phylogenetic relationships^1,5,7^. Clades A and B include five (Allpahuayo, Flexal, Paraná, Pichindé, and Pirital) and seven (Amaparí, Cupixi, Guanarito, Junín, Machupo, Tacaribe, and Sabiá) South American arenaviruses, respectively, regardless of the gene used for phylogenetic analysis^7,8^. Only Oliveros and Latino viruses were identified in Clade C, regardless of the gene sequence used for analysis^5^. Discrepancies were observed for mammarenaviruses indigenous to North America (Tamiami, Whitewater Arroyo, and Bear Canyon viruses) and for a proposed new species from French Guiana. An analysis based on complete sequences confirms that the S RNA genome of these arenaviruses has a chimeric origin, likely a recombination event that occurred in an ancestral virus^9,10^. These viruses form a separate lineage known as Clade A/Rec and are proposed to be named Clade D according to the latest updates in arenaviruses taxonomy^1^.

The Amazon River Basin Region is a vast territory, encompassing nine South American countries: Bolivia, Brazil, Colombia, Ecuador, French Guiana, Guyana, Peru, Suriname, and Venezuela. This region contains the world’s largest tropical rainforest, with a climate characterized by high temperatures and humidity and copious rainfall, and the most varied ecosystem in the world. Favorable conditions exist for the transmission of numerous infectious agents, particularly from increasing contact of the human population to wild interface areas and because of accelerating population growth, environmental, and climate changes^11^. In fact, the Amazon Basin is often regarded as a hot spot for viruses and other pathogens that find optimal conditions to emerge or reinforce their pathogenic potential^12^.

To date, three mammarenavirus were detected in the Brazilian Amazon during surveys conducted in the 1960s: Amaparí virus (*Neacomys guianae*), Cupixi virus (*Oryzomys megacephalus*), and Flexal virus from an unidentified oryzomyini rodent^5,7,13,14^. Here, we report the identification of a novel mammarenavirus in *Neacomys musseri* (Rodentia: Sigmodontinae) from the Amazon Basin Region; we propose that this mammarenavirus be designated as Xapuri virus (XAPV) after the locality where this new virus was detected.

## Results

**Fig. 1:**
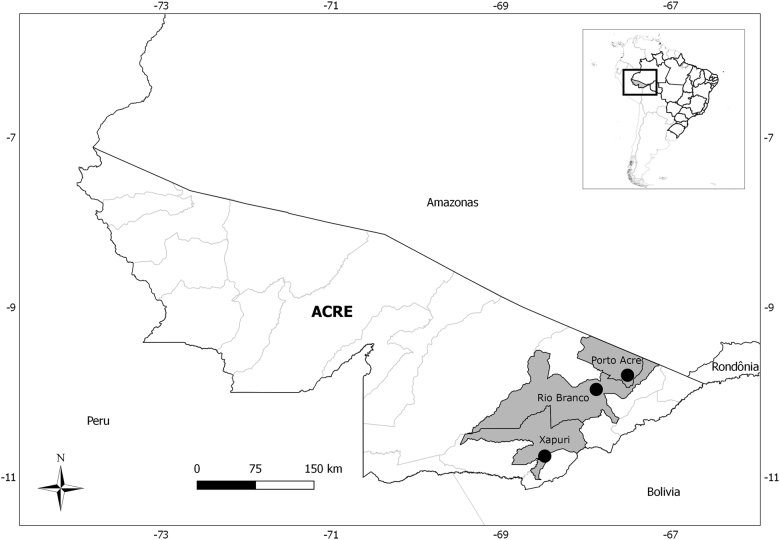
Municipalities of Acre state, Brazil, in which rodents were captured

A total of 49 rodents were analyzed: 22 from Porto Acre, 16 from Xapuri, and 11 from Rio Branco localities; *Neacomys spinosus* (17) and *Oecomys bicolor* (9) were the most abundant (Table 1). Amplification of the partial GPC gene was observed for one *Neacomys musseri* (1/49–2.0%) male collected in 2015, in the Seringal Cachoeira locality, Xapuri municipality (Fig. 1).

**Table 1:** Rodents captured in three localities from Acre state, Brazil, 2015–2016, by species and locality

Species	Number of captured rodents			
	Porto Acre	Rio Branco	Xapuri	Total
*Euryoryzomys macconelli*	—	—	1	1
*Hylaeamys perenensis*	—	—	2	2
*Hylaeamys yunganus*	1	—	1	2
*Mesomys hispidus*	1	—	1	2
*Neacomys musseri*	—	—	4	4
*Neacomys spinosus*	8	3	6	17
*Oecomys bicolor*	7	2	—	9
*Oecomys* sp.	—	—	1	1
*Oligoryzomys microtis*	1	3	—	4
*Proechimys brevicauda*	2	—	—	2
*Proechimys gardneri*	1	1	—	2
*Proechimys simonsi*	—	2	—	2
*Rhipidomys leucodactylus*	1	—	—	1
	22	11	16	49

**Fig. 2:**
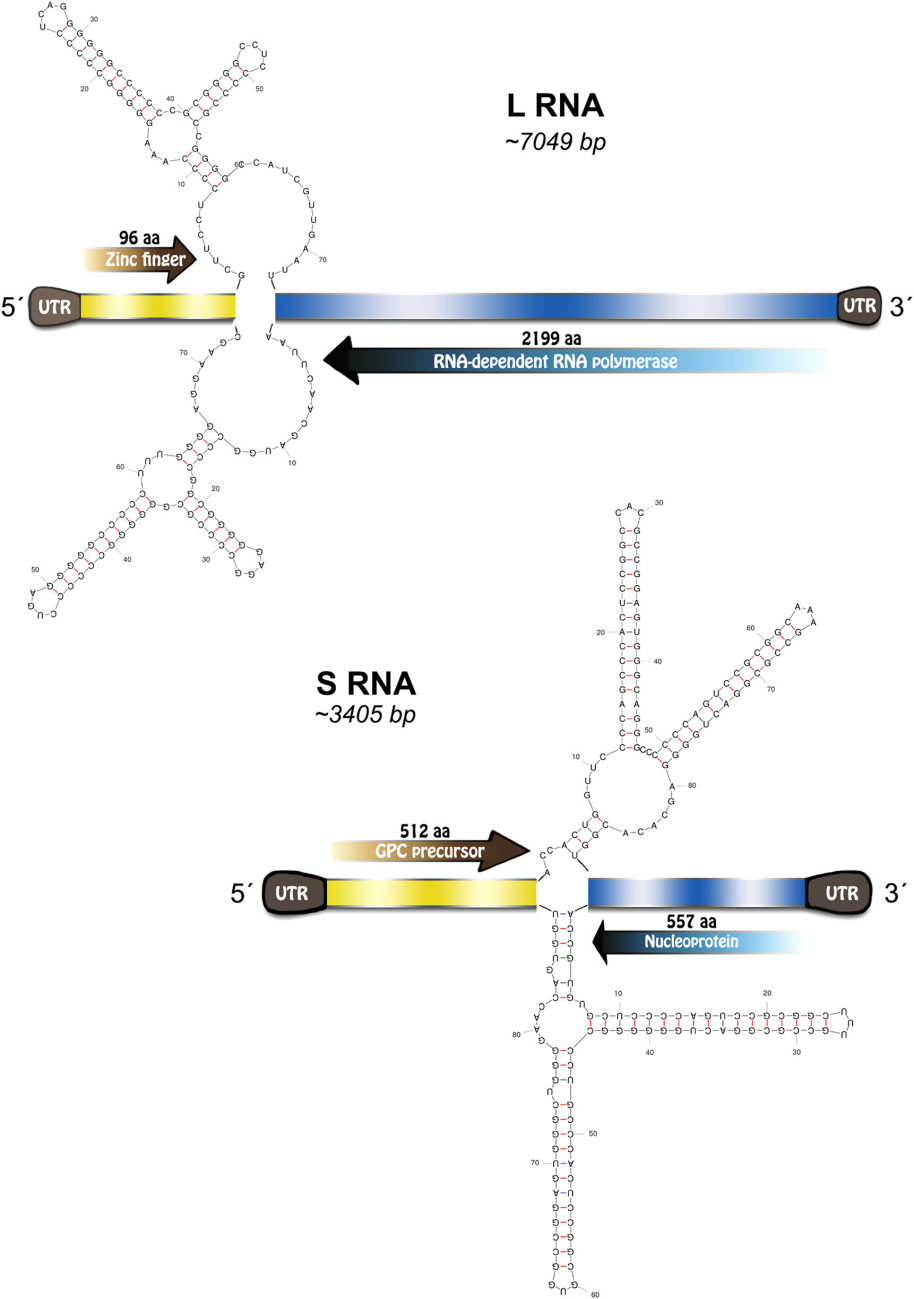
Xapuri mammarenavirus genome organization and potential secondary structure of intergenic regions

Complete genome sequencing of *Neacomys musseri* mammarenavirus included two segments: the L segment (GenBank MG976577) of 7049 nucleotides (nt) and the S segment (GenBank MG976578) of 3405 nt. Each segment encoded two open reading frames (ORFs) in an ambisense organization with an intergenic region of 72 and 88 nt in length containing a predicted hairpin between the ORFs for the S and L segment, respectively. NP, GPC, Z, and L protein lengths were 557 amino acids (aa), 512, 96, and 2199 aa, respectively (Fig. 2). Additional features commonly observed in mammarenavirus genomes include the conservation of the 3′−5′ termini and the presence of an L-domain motif within the Z protein.

Deduced aa and nt sequences from the four proteins and complete S and L segments were compared to those of other representative mammarenaviruses. Nucleotide sequence divergences of >34.8 and >41.2% for the S and L segments, respectively, were found between the *Neacomys musseri* virus and all other known mammarenavirus species, whereas a 38.5% aa sequence divergence was found for the entire NP (Table 2). Pairwise sequence comparison (PASC) was performed on both segments, and our sample was found to be most closely related to Latino (GenBank AF485259) and Oliveros (GenBank NC_010248) viruses demonstrating 62.84–61.32% identity for the S segment, whereas the L segment showed 57.35–55.71% identity with Amaparí (GenBank AY924389) and Guanarito viruses (GenBank NC005082).

**Table 2:** Nucleotide and amino acid identities of XAPV compared with New World representatives of the genus *Mammarenavirus*

Virus species	*p distance*					
	L segment (nt)			S segment (nt)		
		Z (aa)	L (aa)		NP (aa)	GPC (aa)
Allpahuayo	46.7%	50.7%	56.2%	39.9%	45.0%	44.3%
Amaparí	41.2%	48.0%	48.4%	39.8%	40.5%	53.4%
Bear Canyon	47.4%	53.3%	56.9%	42.1%	46.0%	50.6%
Chapare	41.8%	48.0%	48.7%	37.6%	38.7%	46.2%
Cupixi	42.8%	46.7%	48.9%	40.3%	38.9%	51.5%
Flexal	48.1%	52.0%	57.1%	39.9%	44.6%	42.0%
Guanarito	42.8%	40.0%	48.7%	38.8%	39.5%	52.4%
Junín	43.2%	46.7%	50.7%	38.5%	41.5%	48.5%
Latino	42.6%	50.7%	50.2%	35.2%	38.9%	44.8%
Machupo	43.1%	45.3%	48.7%	38.9%	38.5%	47.3%
Oliveros	42,4%	49.3%	48.6%	34.8%	38.5%	34.8%
Paraná	46.3%	52.0%	56.0%	39.6%	45.6%	45.5%
Pichindé	46.8%	56.0%	57.3%	40.8%	45.4%	44.3%
Pirital	46.7%	58.7%	55.7%	39.7%	44.6%	46.6%
Sabiá	41.9%	46.7%	49.6%	37.6%	40.3%	48.3%
Tacaribe	43.0%	46.7%	49.5%	41.5%	42.8%	48.3%
Tamiami	47.8%	48.0%	56.5%	41.6%	47.0%	51.0%
Whitewater Arroyo	47.3%	54.7%	57.2%	42.0%	47.7%	50.8%

*XAPV* Xapuri virus

**Fig. 3:**
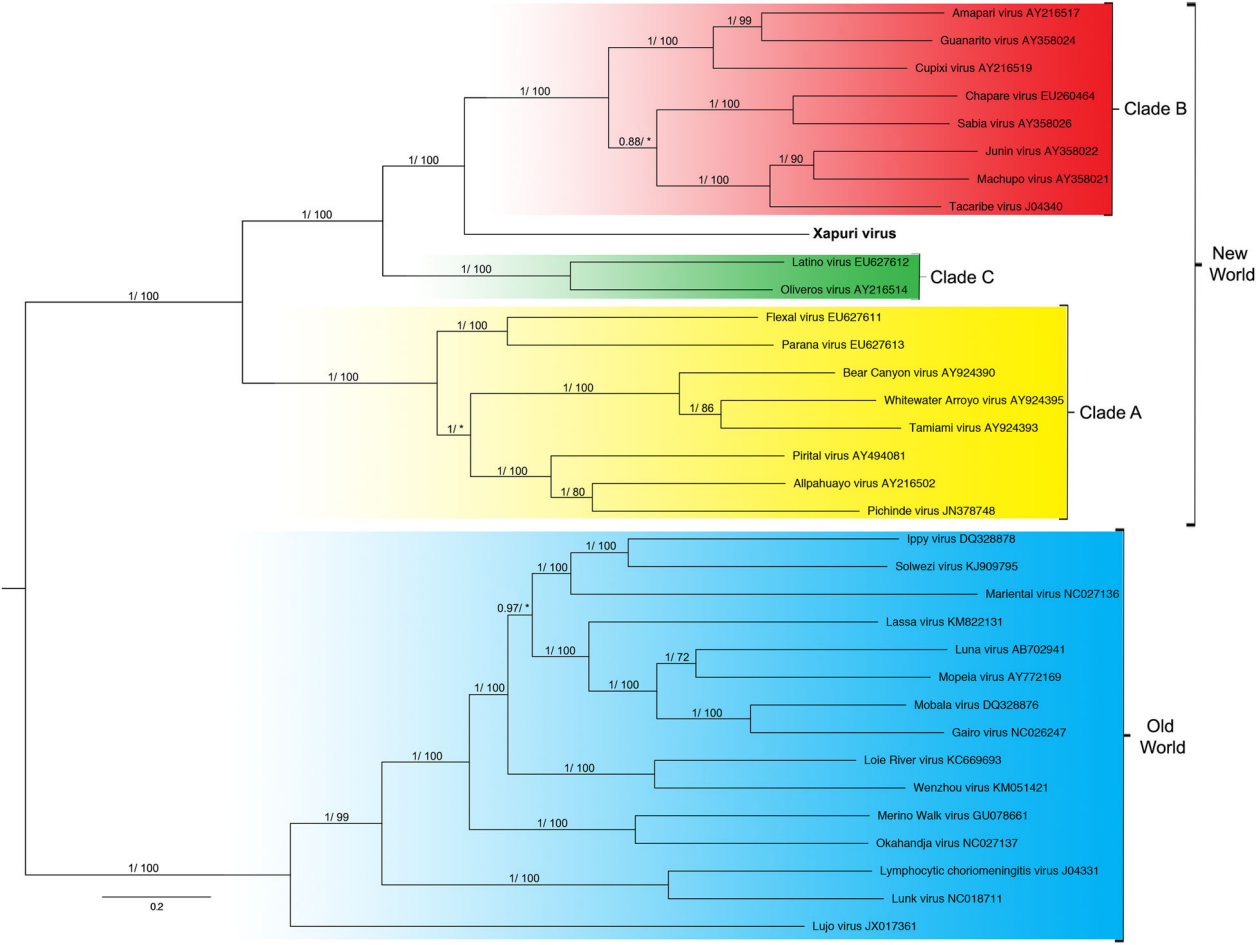
Phylogenetic tree based on the mammarenavirus complete L segments, with ML and Bayesian methods, using the evolutionary model GTR + G + l . Numbers (≥0.7/≥70) above branches indicate node probabilities or bootstrap values (MrBayes/ML). Asterisks indicate values below 0.7/70. Sequences from this study are highlighted in bold

**Fig. 4:**
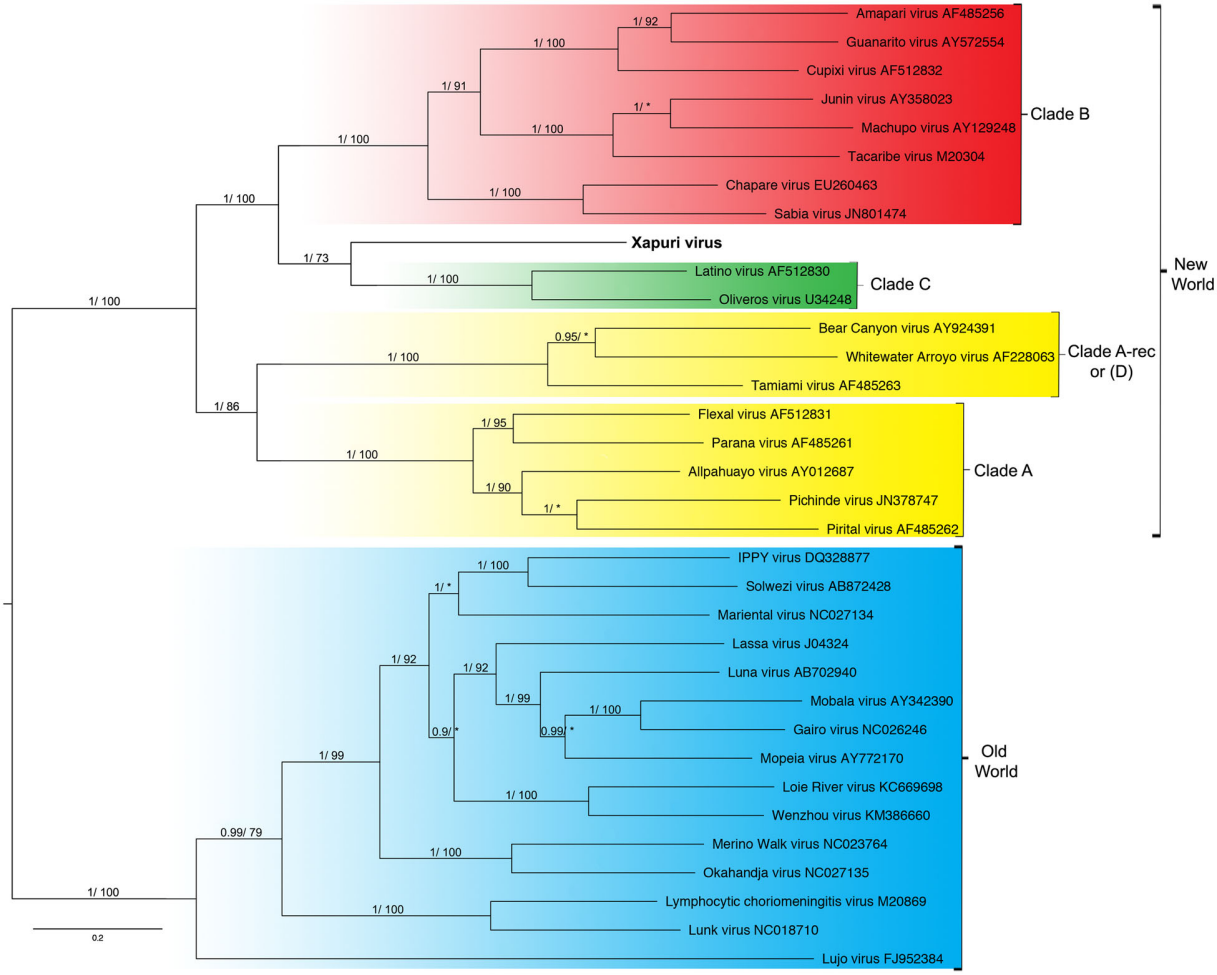
Phylogenetic tree based on the mammarenavirus complete S segments, with ML and Bayesian methods, using the evolutionary model GTR + G + l . Numbers (≥0.7/≥70) above branches indicate node probabilities or bootstrap values (MrBayes/ML). Asterisks indicate values below 0.7/70. Sequences from this study are highlighted in bold

**Fig. 5:**
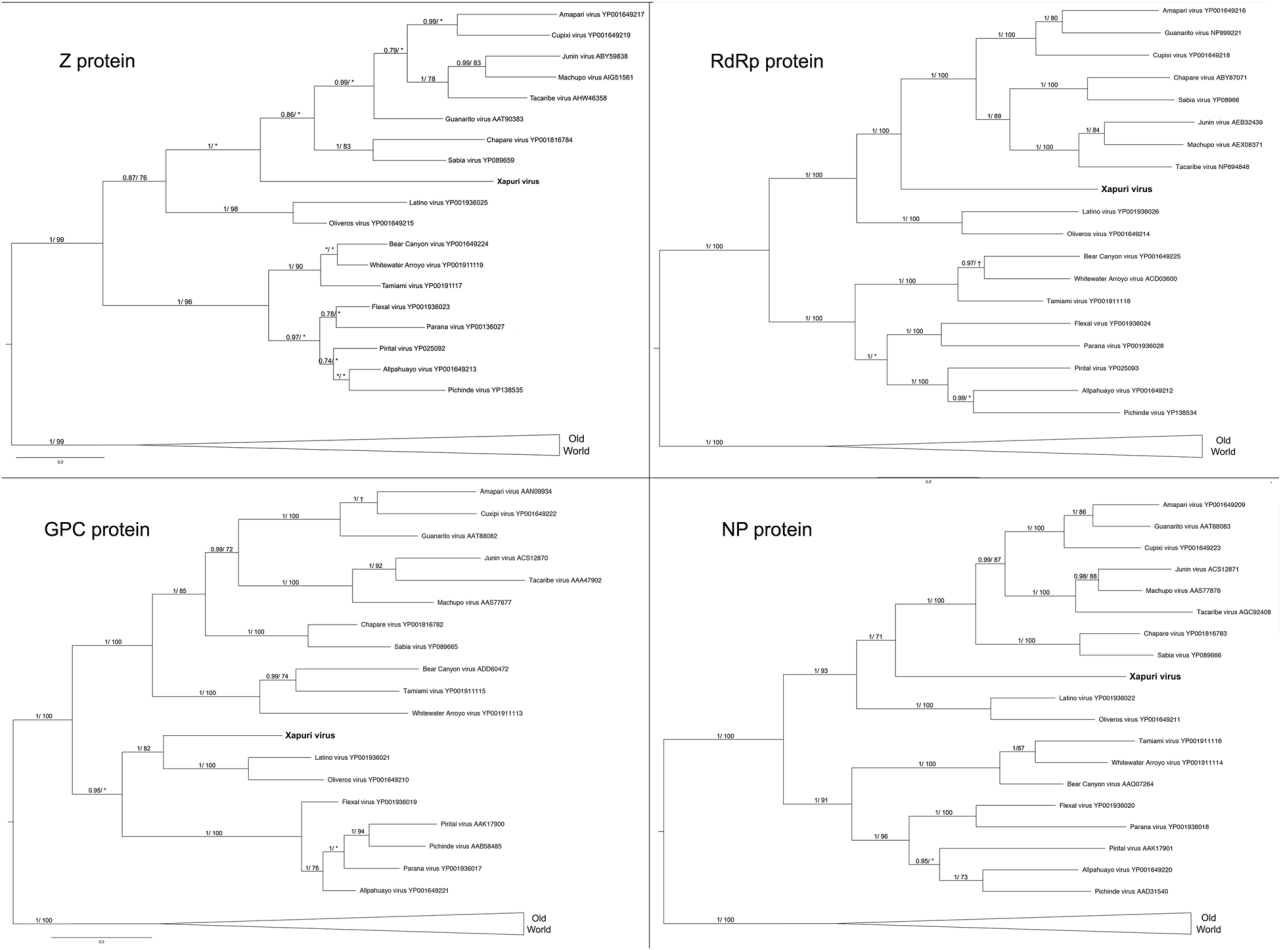
Phylogenetic trees based on the complete NP, GPC, Z, and L mammarenavirus proteins, using ML and Bayesian methods, (Z protein) complete Z, using the evolutionary model RtREV + G + I, (RdRp protein) complete L, using the evolutionary model LG + G + I, (GPC protein) complete GPC, using the evolutionary model LG + G + I, and (NP protein) complete NP, using the evolutionary model. Numbers (≥0.7/≥70) above branches indicate node probabilities or bootstrap values (MrBayes/ML). Asterisks indicate values below 0.7/70. ^†^Exhibited a difference between ML and MrBayes tree-building method topologies. Sequences from this study are highlighted in bold

In the maximum likelihood (ML) and Bayesian phylogenetic analyses for the S and L segments, the Amazonian virus described in this study formed an independent clade closely related to Clades C and B New World mammarenaviruses, respectively (Figs. 3 and 4). Sequences from NP, Z, and L proteins displayed the same topology as the L segment, taking a stem lineage position for New World Clade B viruses (Fig. 5). GPC was the most divergent protein, forming a sister stem lineage clade with New World Clade C viruses (Fig. 5). Bootscan and RDP4 recombination analysis of S and L segment sequences by Simplot revealed no recombination peaks. Reconstructed phylogenetic trees, including the complete nt GPC gene show no alternate clustering of the *N. musseri* virus and Clade C viruses.

## Discussion

The genus *Neacomys* Thomas, 1900 (Cricetidae, Sigmodontinae, Oryzomyini) comprises eight valid species of spiny rats, distributed from Central and South America. They are mainly found in the Amazon region (*N. dubosti*, *N. guianae*, *N. minutus*, *N. musseri*, *N. paracou*, and *N. spinosus*), and only two species do not occur in the Brazilian Amazon (*N. pictus* and *N. tenuipes*)^15,16^. *Neacomys* rodents were identified as important hosts for different rodent-borne viruses in Brazil (Amaparí mammarenavirus) and Peru (Andes orthohantavirus)^13,17^. In fact, *Neacomys guianae* is the host of Amaparí virus, a Clade B mammarenavirus from Amapá state, in the Brazilian Amazon^13^. The detection of a new mammarenavirus in another *Neacomys* reinforces the importance of these rodents in mammarenavirus enzootic cycles, particularly in the Amazon region.

Delineating species in the *Arenaviridae* family follows multiple criteria, including the association with a main host species or group of sympatric hosts, the presence in a defined geographical area, and significant protein sequence differences, such as a variance of at least 12.0% in the aa sequence of the NP compared to that of other species in the genus^1,18^. In addition, a recent update from the International Committee on Taxonomy of Viruses (ICTV)^1^ also includes as classifiable “virus coding-complete genomic sequences for both S and L segments even in the absence of a culturable isolate” and recommends the use of the PASC tool for the assessment of novel arenaviruses. Cut-off values selected for classifying arenaviruses belonging to the same species using this tool are >80.0 and >76.0% regarding nucleotide sequence identity in the S and L segments, respectively^1^. The virus identified in this study from *N. musseri* is the first mammarenavirus detected in this rodent species, the only mammarenavirus isolated from Acre State, and the fourth from the Brazilian Amazon^14,19^. Furthermore, the sequence of this virus also meets the nucleoprotein aa sequence identities and PASC requirements detailed by the ICTV as being novel; thus, we suggest naming it XAPV after the municipality where it was detected. Xapuri in a native language from the Amazon means “river before.” The city received this name because it is located between the Xapuri and Acre rivers. Accordingly, we believe the name is suitable for XAPV’s genetic characteristics, standing as it does between Clade B and Clade C New World mammarenaviruses.

XAPV features are interesting for the Tacaribe virus serocomplex group. The placement of XAPV as a divergent but sister group of Clade C and Clade B mammarenaviruses for S segment and L segments, respectively, could be indicative of reassortment between these clades. Nevertheless, although many studies indicated that viral diversification during arenavirus evolution is due to high mutation rates from a low-fidelity viral RdRp, recombination and reassortment events (as for other segmented RNA viruses), no reassortant mammarenavirus were previously isolated from nature^9,20,21^. This absence of natural arenavirus reassortments was attributed to the superinfection exclusion exhibited by some members of this family in chronic infection models. However, more recent studies demonstrated that acute infection by New World Junín virus failed to down-regulate entry receptors and did not induce superinfection exclusion^22,23^. Additionally, it is noteworthy that arenavirus reassortants have been produced in vitro, and these data indicate that there are restrictions that prevent the recovery of all possible combinations and that only closely related viruses may be able to reassort with one another^20,24,25^. Indeed, recent studies showed that reassortment may be a common event for newly recognized reptarenaviruses^26^. During reassortment events, in which entire genes are exchanged during the swapping of segments, the ORF of the gene and, consequently, the protein integrit, are maintained without changes in ORFs and their encoded proteins as shown for XAPV (Figs. 3–5). Therefore, we propose that XAPV may represent the first identification of a natural reassortant of the *Arenaviridae* family that has arisen from two mammarenavirus groups that are not closely related.

As for recombination events within the S RNA segment of North American arenaviruses (Clade D), reassortment between Clade B and Clade C likely occurred during the early stages of South American mammarenavirus evolution^1,5–7,9,27^. In fact, when we analyze XAPV proteins, a recombination pattern similar to those for Clade D is found. GPC sequence analysis places XAPV in a sister relationship with Clade C, whereas analysis of the N, Z, and L protein sequence data places it in a sister relationship with Clade B^27^.

The generation of reassortant or recombinant arenaviruses requires cells to be simultaneously infected by two or more different viruses. Although coinfections were reported in cell culture, this infection may be less likely to happen in nature^22–24^. Persistent infection of rodent reservoirs is also an important factor that could influence the rate of recombination and reassortment, facilitating coinfection of cells with two different virus^28,29^. Similarly, different mammarenaviruses can sometimes infect the same rodent species, such as Guanarito and Pirital viruses, which were both isolated from *Zigodontomys brevicauda* and *Sigmodon hispidus* in Venezuela^30^. Irwin et al.^31^ suggested that host switching is mainly responsible for arenavirus evolution, which may contribute to coinfection of a single host species with Clade B and Clade C ancestors of XAPV and possibly other related viruses. It is possible that future investigations will reveal new arenaviruses closely related to XAPV and define a new fifth clade within New World mammarenaviruses, composed of chimeric viruses of Clades B and C.

Studies conducted with ML29, an *in vitro* reassortant virus consisting of the Lassa virus S genomic segment and the Mopeia virus L segment, suggest that major virulence factors are located on the L genomic segment^32,33^. In fact, many studies demonstrated key aspects of the L and Z proteins during arenavirus infection^34,35^. A hallmark feature of arenavirus hemorrhagic fevers are the high levels of viremia related to the L protein and its capacity to enhance intracellular levels of replication^36–39^, whereas the Z protein of pathogenic arenaviruses has an immune suppressive function inhibiting interferon responses^40,41^. Although it is not yet clear whether XAPV can cause human infection, its unique features shared with Clade B and Clade C may make it a potential threat to human health.

In conclusion, XAPV may represent a new clade within New World mammarenaviruses and its unique genetic features could shed light onto evolutionary mechanisms of arenavirus evolution and viral diversification. Further studies should be conducted, particularly in the Amazon region, to better understand the epizootiologic aspects of XAPV and its potential to cause human disease, as well as increase the knowledge of the geographic range and genetic diversity of South American mammarenaviruses.

## Materials and methods

### Study area and small mammal trapping

The fieldwork was conducted in Rio Branco (9°58′29″S 67°48′36″W), Porto Acre (09°35′16″S 67°31′58″W), and Xapuri (10°39′07″S 68°30′14″W) municipalities, Acre State, North Brazil (Fig. 1). Rodent sampling was conducted every 6 months between 2015 and 2016, during five consecutive nights for each of the four capture sessions. The capture effort was constant for all capture sessions. We established transects with capture stations setting Tomahawk (Tomahawk Live Trap, Hazelhurst, WI, USA) (40.64 × 12.70 × 12.70 cm^3^) and Sherman (HB Sherman Traps, Tallahassee, FL, USA) (7.62 × 9.53 × 30.48 cm^3^), live traps baited with a mixture of peanut butter, banana, bacon, and oatmeal.

Specimens were captured, anesthetized, and euthanized according to recommended safety procedures and under the Guidelines for the Care and Use of Laboratory Animals, Fundação Oswaldo Cruz, Brazil, License number LW-39/14^42^. Animals were captured with authorization of the Instituto Chico Mendes para Conservação da Biodiversidade (ICMBIO Authorization 13373). Specimens were then measured, weighed, sexed, and identified by cranial morphology/morphometry and karyotyping when necessary. Collected tissues were placed in RNA*later*™ Stabilization Solution (Sigma-Aldrich, St. Louis, MO, USA) and stored at −20 °C. Mammarenavirus-positive rodent specimens were confirmed by molecular analysis (amplification of the cytochrome *b* gene)^43^. Voucher rodent specimens were deposited in the Laboratory of Biology and Parasitology of Wild Mammals Reservoirs collection, IOC/FIOCRUZ, Rio de Janeiro, Brazil.

### Mammarenavirus detection

The total RNA was extracted from liver and kidney tissue fragments using the PureLink Micro-To-Midi total RNA Purification Kit (Invitrogen, San Diego, CA, USA) according to the manufacturer’s protocol. Mammarenavirus detection was performed according to previously described protocols targeting fragments of GPC and NP genes from the S segment of mammarenaviruses^8,44^.

### Metagenomic library preparation

The isolated RNA was depleted of ribosomal RNA using NEBNext rRNA Depletion Kit (Human/Mouse/Rat) (New England BioLabs Inc.) and was cleaned up using a Zymo Clean and Concentrator column (Zymo Research). A 4 μl aliquot of RNA was used to prepare complementary DNA (cDNA) using a Sequence Independent Single Primer Amplification approach adapted from ref^45^. Reverse transcription and second-strand cDNA synthesis were as described. The cDNA amplification was performed using AccuTaq LA (Sigma), in which 5 μl of cDNA and 1 μl (100 pmol/μl) primer B (5′-GTTTCCCACTGGAGGATA-3′) were added to a 50 μl reaction, according to the manufacturer’s instructions. The PCR conditions were as follows: 98 °C for 30 s, 30 cycles of 94 °C for 15 s, 50 °C for 20 s, and 68 °C for 5 min, followed by 68 °C for 10 min. Amplified cDNA was purified using a 1:2 ratio of AMPure XP beads (Beckman Coulter, Brea, CA, USA) and quantified using a Qubit and High Sensitivity dsDNA Kit (Thermo Fisher Scientific Inc.).

### Illumina library preparation and sequencing

An Illumina sequencing library was prepared using the Nextera XT V2 Kit with 1.5 ng of cDNA as the input, following the manufacturer’s instructions. Indices were selected using the Illumina experiment manager software. Samples were multiplexed in batches of a maximum of eight samples per run and sequenced on a 2 × 150 bp paired-end Illumina MiSeq run by the Genomics Services Development Unit, Public Health England.

### Data handling

Reads were trimmed to remove adaptors and low-quality bases, to achieve an average phred score of Q30 across the read, using trimmomatic^46^. BWA MEM v0.7.15^47^ was used to align reads to the *Mus musculus* reference genome (assembly GRCm38.p6). Viral reads were extracted from the fastq files using seq_select_by_id^48^. De novo assemblies were generated using Spades 3.8.2^49^ in combination with SSPACE Standard v3.0^50^. Contigs larger than 1 kb were searched against the National Center for Biotechnology Information (NCBI) protein database using a translated nucleotide query^51^.

Complete coding sequences for both segments of each virus were loaded into the PASC tool and analyzed using the default parameters (https://www.ncbi.nlm.nih.gov/sutils/pasc/viridty.cgi?cmdresult=main&id=448)^52^.

### Phylogenetic analysis

Multiple sequence alignment and comparison of aa were performed using MAFFT version 7 with the E-INS-i algorithm in the Jalview v.4 software program^53,54^. Phylogenetic relationships were estimated with (a) ML phylogenetic inference using PhyML implemented in SeaView v.4 software program^55,56^, and (b) a Bayesian Markov Chain Monte Carlo (MCMC) method implemented in MrBayes v3.2.6^57^. For the Bayesian analyses, we used a mixed aa model of evolution with a γ-shaped distribution of rates across sites. This model allows selection to be integrated across all best-fit models. The MCMC settings consisted of two simultaneous independent runs with four chains each that were run for 10 million generations and sampled every 100th generation, yielding 100,000 trees. After eliminating 10% of the samples as burn-in, a consensus tree was built. Statistical support for the clades was measured by a heuristic search with 1000 bootstrap replicates and the Bayesian posterior probabilities. The best-fit evolutionary model was determined using MEGA version 7, using the Bayesian Information Criterion^58^.

### Detection of recombination

To analyze possible recombination events, a set of 33S and L segment sequences from all mammarenavirus recognized by ICTV (http://ictvdb.bio-mirror.cn/Ictv/fs_arena.htm (accessed 02 February 2018)) were aligned, including the sequence generated in the present study. Sequence alignment was analyzed with Bootscan implemented in Simplot and RDP4 software^59,60^. The sequences for Bootscan analysis were grouped according to the clustering clades in the phylogenetic trees for the L and S segment, and the sequence of the XAPV comprised the query group.
